# Coping With Stress and Burnout Associated With Telecommunication and Online Learning

**DOI:** 10.3389/fpubh.2020.574969

**Published:** 2020-11-11

**Authors:** Nour Mheidly, Mohamad Y. Fares, Jawad Fares

**Affiliations:** ^1^Faculty of Information, Lebanese University, Beirut, Lebanon; ^2^Neuroscience Research Center, Faculty of Medical Sciences, Lebanese University, Beirut, Lebanon; ^3^Faculty of Medicine, American University of Beirut, Beirut, Lebanon; ^4^College of Medical Veterinary & Life Sciences, University of Glasgow, Glasgow, United Kingdom; ^5^Department of Neurological Surgery, Feinberg School of Medicine, Northwestern University, Chicago, IL, United States

**Keywords:** COVID-19, SARS-CoV-2, mental health-state of emotional and social well-being, psychology, students, education-active learning, e-learning, COVID-19 mental health response

## Abstract

The COVID-19 pandemic substantially impacted the field of telecommunication. It increased the use of media applications that enable teleconferencing, telecommuting, online learning, and social relations. Prolonged time facing screens, tablets, and smart devices increases stress and anxiety. Mental health stressors associated with telecommunication can add to other stressors related to quarantine time and lockdown to eventually lead to exhaustion and burnout. In this review, the effects of the COVID-19 pandemic on communication and education are explored. In addition, the relationship between prolonged exposure to digital devices and mental health is studied. Finally, coping strategies are offered to help relieve the tele-burdens of pandemics.

## Introduction

The COVID-19 pandemic has led to a major shift in communication. Advancement in technology played a central role in facilitating this shift. People were pushed toward media applications that enable live connection and interaction between individuals, institutions, firms, and even countries. This mode of communication, done through remote applications, is called telecommunication.

Burnout is a syndrome conceptualized as resulting from chronic workplace stress that has not been successfully managed. Over the past decade, studies exploring stress and burnout in the occupational and educational settings were published extensively. During the COVID-19 pandemic, the workplace setting changed upon lockdown implementation. Appropriate jobs shifted to remote working and telecommunication. In addition, education shifted to online mode and distance learning. Nevertheless, studies exploring stress and burnout associated with the new norm of increased telecommunication are lacking.

Here, we explore the status of communication and learning before and during the COVID-19 pandemic. The intense shift to telecommunication during the outbreak can lead to increased levels of stress and burnout as a result of increased on-screen time. Improving public awareness on the negative consequences of telecommunication and offering practical solutions to cope with its associated mental health challenges is vital to relieve the tele-burdens of pandemics.

## Online Communication in the Era of COVID-19

Before the COVID-19 pandemic, online communication and learning has been growing steadily worldwide, as new digital technologies emerge, and the global adoption of the Internet intensifies. The increased demand for skills that match the rapidly developing digital economy projected that online communication and learning was on track to become a global phenomenon and mainstream by 2025 (1).

The pandemic shifted communication substantially from face-to-face to virtual. Business meetings, academic conferences, education, and governmental management were forced to adapt to the challenges and risks that COVID-19 posed. Telecommunication via Skype, Zoom, FaceTime, and Cisco Webex was key in keeping the educational, economic, and health sectors alive and ongoing during the outbreak. Organizations used tele-detailing by means of social media or email to maintain connectivity and communication (2). In addition, telemarketing witnessed a surge in popularity to promote products and connect with customers (2). This shift further necessitated a change in policies and laws that govern communication in some countries. The UK government, for example, temporarily removed the in-person law for local authorities when holding public meetings, facilitating the conduction of meetings remotely (3).

## Online Learning and the Covid-19 Pandemic

The online learning that we have today dates to the 1990s, when the Internet and World Wide Web started reaching individuals in remote locations and different time zones. This was a major shift from the mid-nineteenth century correspondence courses that started in England and involved sending of hard copy documents between students and university instructors. In the past two decades, advances in communication disrupted the education industry and made online education more feasible technologically, economically, and operationally (4).

The COVID-19 pandemic further accelerated the shift of the education sector toward online learning as gathering of students was forbidden. A high demand for massive online open courses, from providers such as Coursera and edX, was noted during the lockdown (3, 5). In April 2020, people searched for “free online courses” more than one million times. Searches for technology courses that upgrade skills and knowledge, such as Microsoft Excel, Python, and coding, increased by 100% (6). To satisfy the demand, Coursera offered certificates for 115 courses for free (3). Course offerings varied between science, philosophy, history, mathematics, and other topics (3). edX also offered a variety of free online courses in partnership with various institutions to teach the history of pandemics, the actions that should be taken during pandemics, the available treatments for the virus, and how to manage during pandemics (5).

Extended protective measures forced most schools and universities to close their campuses until better control of the pandemic is achieved. The University of Cambridge, for example, announced that the 2020/2021 academic year will be fully online (7). Colleges in the US responded to the pandemic gradually. Touro College and Stanford University were the first to announce their shift to online learning in March 2020. Later, 1,400 private and public educational institutions joined, and announced their transition to online learning (8). Some institutions were reluctant to fully endorse online education straightaway. Harvard University officials, for instance, announced that only some of its schools, such as the schools of design, divinity, and public health, will conduct on-line classes during the Fall 2020 semester (9). In China, the Spring 2020 semester was postponed. To cope with the challenges, the Chinese Ministry of Education issued a notice stating that elementary and middle schools should be held online. Additional web-based and television resources were provided by the government to specific rural areas where there is a lack of network coverage. Special programs were provided for students to increase their health and public safety education and help them cope with epidemics (10). In Georgia, learning shifted online in the Spring 2020 semester. Various facilities were provided to schools, teachers, and students by Georgia's Ministry of Education, Science, Culture and Sports. A total of 2,086 public schools were equipped with the Microsoft Teams platform and accounts for students and teachers were registered to be able to host virtual classes (11). In Germany, the COVID-19 pandemic highlighted hidden challenges in the educational system, which is lagging when it comes to digital learning (12). Officials have lately started to provide schools and teachers with web services and email accounts (13). Nevertheless, teachers lack the training needed for proper online education and are uninformed about technologies and the German digital infrastructure (12). In addition, several universities were shocked to know that their system is limited with respect to online library access, or the number of simultaneous teleconference participants (12).

## Telecommunication, Wellbeing, and Mental Health

The surge in telecommunication led people to spend more time facing screens, tablets, and smart phones. Previously, the increase in exposure to smart devices and screens has been reported to increase stress and burnout levels. Stress is an emotional, physical, or mental reaction that causes tension (14). It can result from social, environmental, or psychological situations. Burnout is a mental health state that results from work-related distress, involving a continuous reaction to persistent interpersonal stressors. The major factors contributing to burnout are overwhelming exhaustion, feelings of cynicism, and detachment. In addition, a sense of ineffectiveness and lack of accomplishment may ensue (15). Appropriately, occupational burnout is conceptualized as a breakdown in the relationship between people and their work (16).

The relationship between the use of smart devices and stress and burnout has been a topic of interest for researchers worldwide (17). It is often contended that exposure to computer and smartphone screens is associated with a plethora of stress-related symptoms (18). These may appear in the form of psychological, cognitive, or musculoskeletal impairments, and may take a toll on the individual's quality of life and daily function (19).

Few studies have addressed how stress due to smart devices correlates with social and demographic variables. Some gender-focused studies exploring psychological effects of prolonged use of smartphones reported more depressive symptoms and sleep disturbances among females than males (17, 18). Studies on personality traits showed that extroverted personalities were associated with telecommunication burnout, whereas introverts were found to face stresses resulting from telecommunication more easily (20). Age and time also seem to have an effect. Electronic media usage at night among adolescents was associated with decreased sleep duration and increased depressive symptoms (18). The duration of exposure further impacts levels of stress and anxiety. Visnjic et al. explored smart device use among university students and indicated that the intensity and modality of smart device use can influence the development of mental health problems in that population (21). Particularly, it was shown that anxiety is more common in younger students, those who send more text messages, and those who browse the internet less frequently (21). Stress was found to be more prevalent in students who spend longer times per day talking on the phone (21). Khouja et al. further confirmed that increased computer usage among teenagers is associated with increased anxiety levels (22). Madhav et al. showed that increased online activity amongst a cohort of 3,201 US students was associated with moderate-to-severe depression (23). Excessive use of e-mails might also be an antecedent for employee burnout. E-mail overuse can cause information overload, while the stress related to continuously answering e-mails may be an antecedent of burnout (24–26).

It is worth noting that the effects of prolonged exposure to telecommunication can also affect physical health. Observing screens and hunching over smartphones for extended periods of time leads to physical harm. Fares et al. found neck pain to be a prominent problem among adolescent and pediatric users, mainly due to the prolonged and distorted positioning when using these devices (27). Specifically, bending the neck when using digital screens and smartphones may progressively lead to stresses on the cervical spine; a condition known as “iHunch.” It may also strain the ligaments, muscles, and tendons of the vertebral column (27, 28).

## Other Stressful Factors During Pandemics

Pandemics are often associated with a state of stress and panic. Accordingly, strain resulting from telecommunication can accumulate with other stressors to lead to exhaustion, anxiety, and burnout. During the COVID-19 outbreak, imposed lockdowns and compulsory quarantines increased levels of tension (29). The inability to socialize, attend gatherings and interact with others enhanced separation anxiety, boredom, and suicidal thoughts, and as such, these emotions were reported more often (29). Brooks et al. showed that people who spent more than 10 days in quarantine were more likely to report posttraumatic stress symptoms (29).

The elderly, teens, healthcare providers, and individuals with pre-existing mental health problems became more susceptible to stress and burnout. Symptoms in elderly manifested as changes in eating habits, disturbed sleep cycles, increased intake of tobacco and alcohol, and difficulty in concentrating (30). The closing of schools and educational institutions and the shift in learning to online methods disrupted the lives of students (31, 32). Exams were postponed or canceled, graduation ceremonies were halted, and learning objectives were shifted. This took a heavy toll on the psychological well-being of students worldwide. The shift in utilization of healthcare resources toward disease containment and prevention sidelined many medical conditions in the process (33). In the case of patients with mental health problems, this manifested as an aversion toward seeking help, an adjournment of psychiatric appointments, or a shortage in supplying mental health medications (33).

The lockdown negatively affected many sectors through delaying promotions, cutting wages, and/or job termination. Many institutions were forced to limit their working capacity or stop working completely, and this had a negative socioeconomic impact on employees, employers, and national economies (34). Subsequently, pressure and anxiety levels increased among affected individuals, who had to worry about both the pandemic and the burden of providing for themselves and their families during challenging times.

## Recommendations

**Figure 1:**
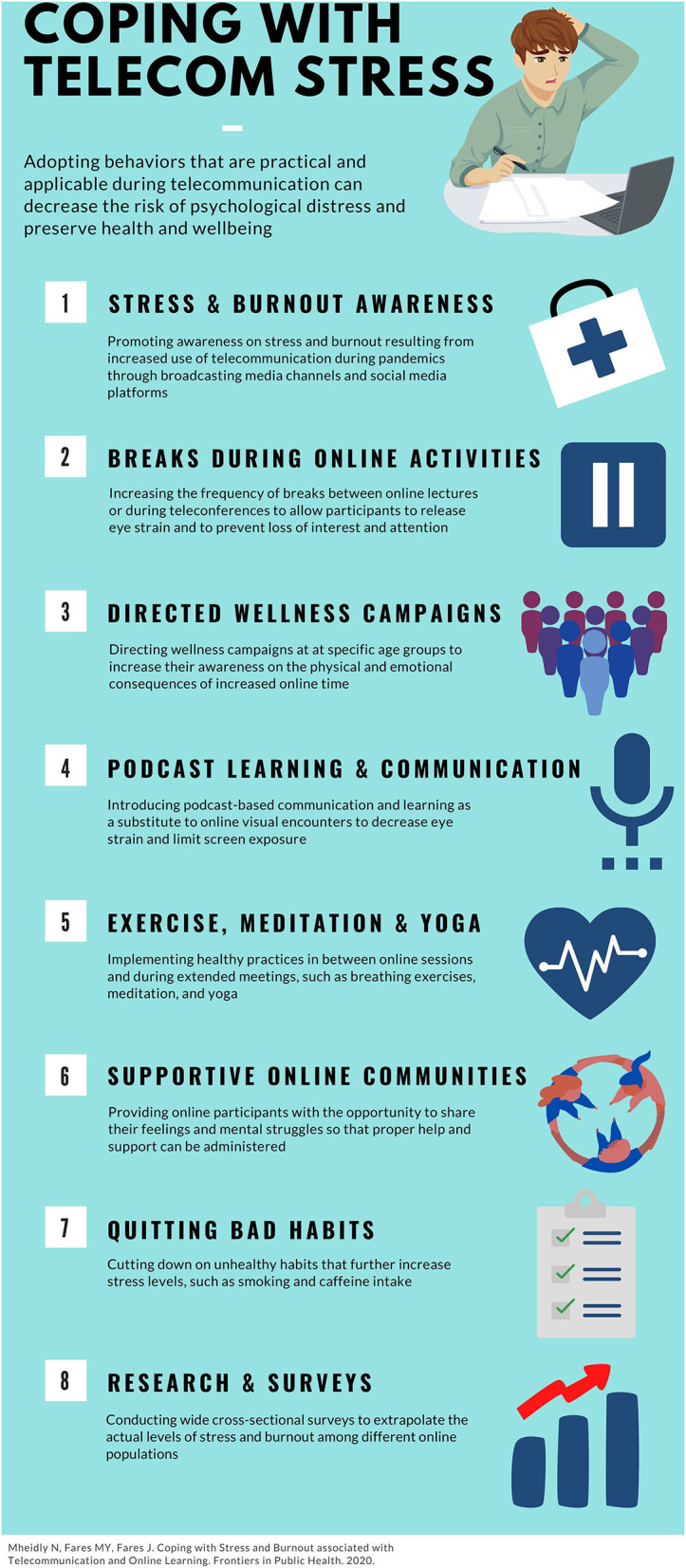
An infographic presenting the coping strategies to be adopted during extended online activities and learning sessions.

Adopting coping strategies that are practical and applicable during online sessions can decrease the risk of psychological distress and preserve health and well-being (Figure 1).

As such, several measures must be taken to increase public mindfulness regarding the psychological repercussions of telecommunication. In addition, health strategies need to be adopted to help the public cope with rising stress and burnout levels during pandemics. Here, we suggest the following:

**1. Promoting awareness on stress and burnout resulting from increased use of telecommunication during pandemics through broadcasting media channels and social media platforms**.

Conventional media must acknowledge stress and burnout related to the pandemic and provide evidence-based data on prevalence and coping mechanisms to the general public. Social media will eventually pick up this information and will facilitate its quick sharing amongst the public (35).

**2. Increasing the frequency of breaks between online lectures or during teleconferences to allow participants to release eye strain and to prevent loss of interest and attention**.

Digital eye strain is an emerging public health issue that results from the continuous use of digital devices. Altered blinking patterns, excessive exposure to intense light, closer working distance, and smaller font size are factors associated with telecommunication through tablets and digital screens that can lead to eye strain and its subsequent effects. Maintaining a normal blinking rate and using artificial tears can help in the management of digital eye strain (36). Increasing breaks between online sessions can also release accumulating tension and maintain interest and attention.

**3. Directing wellness campaigns at students to increase their awareness on the physical and emotional consequences of increased online time**.

Leading awareness campaigns directed at specific age groups while considering cultural and ethnic differences can help increase compliance to healthy online habits. Campaigns presented by role models can grab attention and induce behavioral change. Television personalities, movie stars, and famous athletes have all been shown to impact certain decisions of adolescents (37). Having these entertainers or athletes endorse awareness campaigns may influence people's attitudes and intentions when it comes to telecommunication.

**4. Introducing podcast-based communication and learning as a substitute to online visual encounters to decrease eye strain and limit screen exposure**.

Podcasts are episodic digital audio recordings that are downloaded through web syndication or streamed online (38). In medical education, they have gained widespread popularity compared to other media resources (39), as they possess the potential to facilitate communication between researchers, policymakers, and the public. The creation of a learning podcast is an attractive way to provide asynchronous education because the barrier to entry is low and the resources needed are readily available and inexpensive (40). More importantly, podcasts substitute eyes for ears, easing the strain and stress that can result from prolonged visual fixation on screens and tablets.

**5. Implementing healthy practices in between online sessions and during extended meetings, such as breathing exercises, meditation, and yoga**.

There is growing evidence that yoga is an effective multi-component health intervention that reduces stress, increases physical activity and improves well-being (41–45). Clinical studies provide preliminary support for the effectiveness of yoga as an adjunct treatment for a range of chronic conditions such as depression (46, 47) and anxiety (48). In addition, psychological mechanisms such as enhanced self and body awareness, coping, mindfulness, self-compassion, and social connectedness may underlie healthier lifestyle choices and more adaptive responses to stressors (49). Therefore, adopting such healthy practices can help in coping with stress and burnout resulting from telecommunication.

**6. Providing online participants with the opportunity to share their feelings and mental struggles so that proper help and support can be administered**.

Professionals partaking in online communities must be proactive in using online platforms to share their feelings and experiences with telecommunication. Through these online communities, users might express emotions and provide tips on how they cope with stress and burnout resulting from prolonged online activity. For example, preschool teachers, using online learning communities, improved their knowledge on mental health issues by sharing and discussing experiences related to mental health with others in these online spaces (50).

**7. Cutting down on unhealthy habits that further increase stress levels, such as smoking and caffeine intake**.

An online survey of 957 smokers in the Netherlands reported that 18.9% reported smoking more in May 2020 (51). Severely stressed smokers were even more likely to have increased smoking behavior during the pandemic. Smoking has been linked to depression, anxiety, suicidal thoughts, and weak learning outcomes (52). Caffeine intake may also increase mental health disorders, such as anxiety (53). Cutting down on these bad habits can decrease their additive effect on online-associated stress and burnout.

**8. Conducting wide cross-sectional surveys to extrapolate the actual levels of stress and burnout among different online populations**.

Studying epidemiological patterns and trends related to telecommunication and its associated stress and burnout can help us decipher the risk factors and protective mechanisms that can be studied and highlighted. Furthermore, efforts should be dedicated to fund health communication research (54–56). This will contribute to the advancement of better ways of communication between the different components of the health sector and, subsequently, improve public health and individual well-being.

## Conclusion

Recognizing stress and burnout resulting from telecommunication during pandemics is necessary to develop effective mitigation strategies. Research conducted on stress and burnout during the COVID-19 pandemic mainly focused on physicians, nurses, and other healthcare workers (57–61). Screening and surveying studies exploring stress and burnout levels among the general population or other vulnerable groups are lacking. Despite many reports of association between the use of smart devices and mental and psychological consequences, the evidence to this claim remains equivocal. Some studies in the literature report benefits garnered from the use of cellphones and describe it as a tool for coping with stress rather than causing it (62, 63). Consequently, it is not possible to summarize the relationship between smart device use and stress or draw conclusions on their association. Smart devices have grown in popularity to become an integral part of every household, and accordingly, research on its effects is necessary to be able to reap the benefits of this technology in a safe and healthy manner.

## Author Contributions

NM, MF, and JF conceived the study, collected data, and prepared data presentation. All authors wrote, reviewed, and approved the final version of the manuscript.

## Conflict of Interest

The authors declare that the research was conducted in the absence of any commercial or financial relationships that could be construed as a potential conflict of interest.
